# Frequent Detection of Anti-Tubercular-Glycolipid-IgG and -IgA Antibodies in Healthcare Workers with Latent Tuberculosis Infection in the Philippines

**DOI:** 10.1155/2012/610707

**Published:** 2012-04-05

**Authors:** Umme Ruman Siddiqi, Prisca Susan A. Leano, Haorile Chagan-Yasutan, Beata Shiratori, Hiroki Saitoh, Yugo Ashino, Yasuhiko Suzuki, Toshio Hattori, Elizabeth Freda O. Telan

**Affiliations:** ^1^Division of Emerging Infectious Diseases, Graduate School of Medicine, Tohoku University, Sendai, Miyagi 980-8574, Japan; ^2^STD AIDS Cooperative Central Laboratory, San Lazaro Hospital, Manila, Philippines; ^3^Department of Global Epidemiology, Research Centre for Zoonosis Control, Hokkaido University, Sapporo, Japan

## Abstract

Anti-tubercular-glycolipid-IgG (TBGL-IgG) and -IgA (TBGL-IgA) antibodies, and the QuantiFERON-TB Gold test (QFT) were compared in healthcare workers (HCWs, *n* = 31) and asymptomatic human immunodeficiency virus-carriers (HIV-AC, *n* = 56) in Manila. In HCWs, 48%, 51%, and 19% were positive in QFT, TBGL-IgG, and -IgA, respectively. The TBGL-IgG positivity was significantly higher (*P* = 0.02) in QFT-positive than QFT-negative HCWs. Both TBGL-IgG- and -IgA-positive cases were only found in QFT-positive HCWs (27%). The plasma IFN-*γ* levels positively correlated with TBGL-IgA titers (*r* = 0.74, *P* = 0.005), but not TBGL-IgG titers in this group, indicating that mucosal immunity is involved in LTBI in immunocompetent individuals. The QFT positivity in HIV-AC was 31% in those with CD4+ cell counts >350/*μ*L and 12.5% in low CD4 group (<350/*μ*L). 59 % and 29% were positive for TBGL-IgG and -IgA, respectively, in HIV-AC, but no association was found between QFT and TBGL assays. TBGL-IgG-positive rates in QFT-positive and QFT-negative HIV-AC were 61% and 58%, and those of TBGL-IgA were 23% and 30%, respectively. The titers of TBGL-IgA were associated with serum IgA (*P* = 0.02) in HIV-AC. Elevations of TBGL-IgG and -IgA were related to latent tuberculosis infection in HCWs, but careful interpretation is necessary in HIV-AC.

## 1. Introduction

Although the incidence of tuberculosis has been falling since 2002, there were still 8.8 million incident cases of TB, 1.1 million deaths from TB, and an additional 0.35 million deaths from HIV-associated TB in 2010 [1]. The high rate of latent TB infection (LTBI) is one of the factors that make it difficult to achieve global control and eliminate TB [2]. The recent introduction of the immune-based interferon-*γ* release assay (IGRA) made a great impact on facilitating the diagnosis of LTBI [3] and clarified the high rate of infection in TB-high-risk populations including healthcare workers (HCWs) [4]. Attempts to detect LTBI in HIV-infected individuals were also facilitated by the development of IGRA, although their higher rates of pseudonegative IGRA response due to low CD4+ T cell counts and diminished Th1 immunity cannot be ignored [5]. Trehalose 6,6-dimycolate (TDM), which constitutes a major part of the mycobacterial cell wall, was identified as the most immunogenic glycolipid and is produced predominantly by virulent MTB as well as by atypical mycobacteria [6]. Tubercular-glycolipid antigen (TBGL) consists of TDM purified from virulent mycobacterial strain H37Rv [7, 19]. The immunoglobulin-G to tubercular-glycolipid antigen (TBGL-IgG) has been proposed to be a useful marker for the serodiagnosis of active pulmonary tuberculosis (PTB) in Japan [7]. However, frequent elevated titers (17%) were also found in healthy elderly control people (age > 40 yrs) in the same study, and the possibility of LTBI was suggested by Maekura and colleagues [7]. Although IgA antibody to TBGL antigen (TBGL-IgA) was not evaluated earlier as a biomarker, strong association was revealed between the TBGL-IgG and-IgA titers in PTB cases [8]. Frequent positivity for TBGL-IgG (46%) and -IgA (36%) in healthy adults was also observed in our very recent study in Thailand, a TB-endemic country [9]. The TBGL-IgG-positive responses were not related to BCG vaccination [10]. Since both cellular-mediated and humoral immunity are necessary for an effective immune response against MTB, we aimed to clarify the relationship between the TBGL-IgG and -IgA responses with QuantiFERON-TB Gold In-Tube (QFT) assay system, in healthcare workers (HCWs) in a hospital of the Philippines.

Infection of human immunodeficiency virus (HIV) has substantially boosted the occurrence of tuberculosis (TB) disease worldwide [1]. The devastating association between HIV and TB is responsible for one of four TB-related deaths [11]. The East-Asian countries are predominantly TB endemic [1]. Similarly to Sub-Saharan Africa, the rapid, progressive increase of HIV infections in East-Asian countries may further accelerate TB infection in HIV/AIDS patients [12]. To clarify how HIV infection may alter immune responses in LTBI, newly diagnosed, asymptomatic, non-TB HIV-infected individuals were studied.

To understand the health condition of the individuals, we measured two TB-related biomarkers. Leptin, a cytokine-like hormone produced by bronchial epithelial cells and type II pneumocytes in addition to adipose tissue, exhibits a Th1-bias immune response [13]. Osteopontin (OPN) is a member of extracellular matrix proteins that is synthesized within the immune system by activated T cells, NK cells, dendritic cells, and macrophages. Involvement of OPN in Th1 immune responses has been reported [14]. OPN deficiency was found to be associated with the dissemination of mycobacterial disease, and its expression correlated with an effective immune and inflammatory response against mycobacteria in rodents as well as in human [15, 16]. Elevated levels of circulatory plasma OPN [17] and low levels of leptin [18] were reported to be associated with active tuberculosis; these biomarkers served as a negative evidence of active disease.

## 2. Materials and Methods

### 2.1. Study Subjects

 A case-control study was conducted between March and October of 2010 in adult participants (age > 18 years) in the Philippines. Thirty-one healthy, adult healthcare workers (HCWs) without any concomitant symptoms or chest radiographic findings relevant to active TB and who had negative HIV serology were recruited from San Lazaro Hospital (SLH), Manila, Philippines. Fifty-six newly diagnosed, asymptomatic HIV carriers (HIV-AC) without any clinical symptoms relevant to tuberculosis were randomly selected from among patients receiving care at the outpatient department of the SLH. None of the subjects took any anti-HIV therapy. Subjects with AIDS-defining events, currently active tuberculosis, or any symptoms relevant to tuberculosis, other than active pulmonary diseases, underlying malignancy or metabolic disorders were excluded from the study. The exclusion criteria for active tuberculosis were based on both clinical findings and chest X-ray (CXR) findings in the HCWs. The study was approved by the ethics committee of SLH and the Tohoku University Hospital. We obtained written informed consent from all the participants. Three mL of blood was obtained directly (one mL in each tube) from each participant to perform the QFT assay. Simultaneously, plasma was separated from blood by centrifugation after treatment with EDTA and was aliquoted to CryoTubes for storage at −80°C until further utilization. All the procedures were conducted in accordance with the Helsinki declaration.

### 2.2. TBGL-Antibody Assay

 TBGL-IgG antibody and -IgA antibodies were measured using the Determiner TBGL Antibody ELISA kit (Kyowa Medex, Tokyo, Japan), an in vitro enzyme-linked immunosorbent assay for the quantitative measurement of anti-TBGL-IgG and -IgA in plasma. This assay employs glycolipid antigens purified from *M. tuberculosis* H37Rv (TBGL antigen) coated on a 96-well plate. The details of the assay were described in our previous study [8]. The antibody titers for TBGL-IgG and -IgA were expressed as U/mL. Positive TBGL-IgG titers were determined according to the cutoff index proposed by Kishimoto et al. [19]. The samples were classified as positive when the serum levels of anti-TBGL-IgG were ≥2 U/mL. An arbitrary cutoff value of ≥2 U/mL for TBGL-IgA was used according to the unpublished data of our previous study [8].

### 2.3. QuantiFERON-TB Gold In-Tube (QFT)

 The QFT test was performed using fresh whole blood in accordance with the manufacturer's instruction (Cellestis, Australia). The results were interpreted using specific software provided by Cellestis. The result was scored positive if the IFN-*γ* concentration in the tube TB-specific antigen containing was >0.35 IU/mL after subtracting the value of the nil control (IFN-*γ*-nc) and at least >25% of NC value. If the net IFN-*γ* response (TB Ag minus nil) was <0.35 IU/mL for the antigens and the response to the mitogen-positive control was >0.5 IU/mL, the response was considered as test negative. An intermediate result was recorded if the net IFN-*γ* response was <0.35 IU/mL for the antigen and <0.5 IU/mL for the mitogen and/or was above 8 IU/mL for the NC.

### 2.4. Leptin and OPN Elisa Assay

 Plasma leptin levels were determined by sandwich ELISA using Quantikine Human Leptin Immunoassay kit (R&D Systems) for the quantitative determination of the human leptin concentrations in plasma according to the manufacturer's guidelines. Plasma OPN concentrations were determined using Human OPN Elisa kit (Immuno-Biological Laboratories, Takasaki, Japan) according to the manufacturer's guidelines, and values were expressed as ng/mL.

### 2.5. Clinical Data

 We measured different laboratory markers including complete red blood cell counts, the number of white blood cells with their differential counts, levels of hemoglobin, and serum levels of IgG and IgA. The number of CD4+ T cell counts and HIV RNA load of HIV-AC were also determined.

### 2.6. Statistical Analysis

The data of quantitative variables are summarized as median and range. Categorical variables were computed as frequency and percentage. The data were analyzed using Stat Flex software, version 5 (Artech Co., Ltd: http://www.statflex.net/index.html) and Statcel 2 (OMS Publishing Inc. Saitama, Japan). The ability of each single marker to discriminate HIV from HCW by receiver operating characteristic (ROC) curve and the area under curve (AUC) was also analyzed. The percentage of overall agreement between QFT and TBGL-IgG/IgA ELISA assays was calculated, and a Cohen's Kappa coefficient was used to assess the level of agreement. The significance of association for categorical variables was estimated by Fisher's exact test, whereas correlations between continuous variables were evaluated by Spearman's rank correlation coefficient. The differences in significance between continuous variables were compared by the Mann-Whitney *U* test. A 2-tailed *P* value of <0.05 was considered significant.

## 3. Results

### 3.1. Characteristics of Study Participants

A total of 31 HCWs and 56 newly diagnosed HIV-AC were enrolled in the current study. Basic demographic and clinical characteristics of the study participants are shown in Table 1. The participating HIV-AC were relatively young (*P* = 0.03) with a significant male predominance (*P* < 0.0001) compared to the HCWs. Although lymphocyte counts were comparable between the two groups, total counts of WBC, neutrophils, and monocytes were significantly lower in HIV-AC.

### 3.2. QFT and TBGL-Antibody Assays in HCWs

Forty-eight percent (15/31) of the HCWs showed positive reactions in the QFT assay indicating high incidences of LTBI (Table 1). The median age of the QFT-positive responders from among the HCWs were significantly higher than those of the QFT-negative group (*P* = 0.002). TBGL-IgG and TBGL-IgA were positive in 51% and 19% of HCWs, respectively (Table 1).

**Figure 1:**
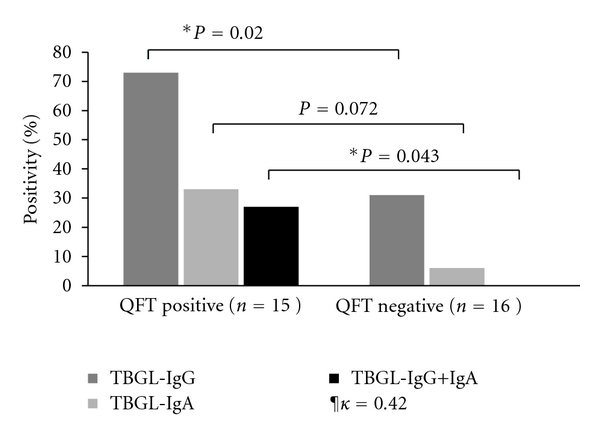
Positivity percentage of TBGL-IgG/IgA assay in QFT-positive/negative healthcare workers. The level of agreement between QFT and the TBGL-Ab assay was measured by Cohen's kappa (*κ*). ¶*κ* = 0.42; overall agreement 71%; 95% confidence interval: 0.1~0.73. *Significant difference (*P* < 0.05).

Eleven of 15 (73%) QFT-positive HCWs had positive TBGL-IgG responses (categorical agreement 73%), whereas 5 of 16 (31%) QFT-negative subjects had positive TBGL-IgG responses (categorical agreement 68.7%). The overall *κ* value was 0.42, indicating a moderate association between the two assays (overall agreement: 71%; 95% CI: 0.10~0.73). The TBGL-IgG-positive proportions were also significantly different between QFT-positive and QFT-negative groups of HCWs (*P* = 0.02). Although the number of positive TBGL-IgA responders was small in HCWs and failed to show any significant difference (*P* = 0.072), the TBGL-IgG+IgA double-positive response was shown only by QFT-positive HCWs and none of the QFT-negative HCWs had double-positive reactions (*P* = 0.043) (Figure 1) (Table 2).

**Figure 2:**
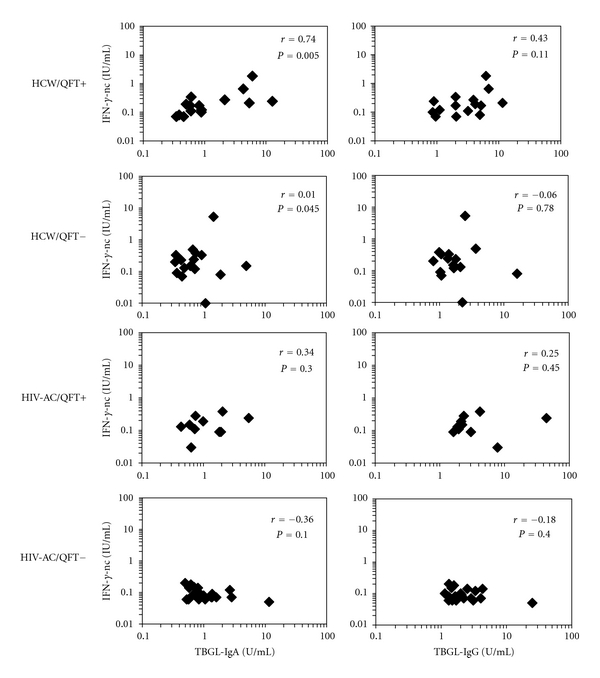
Correlations between TBGL-IgA or TBGL-IgG titers and IFN-*γ* concentrations measured in nonstimulated QFT-plasma samples (IFN-*γ*-nc) in QFT-positive/QTF-negative healthcare workers (HCWs) and asymptomatic HIV carriers (HIV-AC). The only significant positive correlation was observed between the IFN-*γ*-nc concentrations and TBGL-IgA titers in the QFT-positive HCW group (*r* = 0.74, *P* = 0.005).

In addition, significant positive correlation was observed between the concentrations of IFN-*γ*-nc and TBGL-IgA titers in the QFT-positive group (*r* = 0.74, *P* = 0.005) (Figure 2), but not in the QFT-negative group. There was no such association between IFN-*γ*-nc and TBGL-IgG levels in HCWs, although a tendency for a positive correlation was observed in the QFT-positive HCWs (*r* = 0.43, *P* = 0.11) (Figure 2). No association was observed in the net IFN-*γ* concentrations in antigen-stimulated QFT-plasma with TBGL-IgG or -IgA titers (data not shown). The plasma levels of OPN and leptin were not different between QFT-positive and QFT-negative HCWs (Table 2).

### 3.3. QFT and TBGL-Antibody Assays in HIV-AC

As shown in Table 1, only 13 of 56 (23%) HIV carriers showed positive reactions by QFT assay. The rate of positivity was closely associated with high median CD4+ T cell counts (*P* = 0.012) and younger age (*P* = 0.036) (Table 2). Seven of 56 (12.5%) HIV-AC who had lower mitogen responses (IFN-*γ* concentrations: median: 1.78 IU/mL; range: 0.38~6.73 IU/mL) than the rest (>10 U/mL) had negative responses by QFT assay. Their median CD4+ T-cell counts were 60/*μ*L (range: 43~425/*μ*L) (data not shown). Thirty-three of 56 (59%) and 16 of 56 (29%) HIV-AC were attributed with positive TBGL-IgG and TBGL-IgA responses, respectively (Table 1). The positive proportions of TBGL-IgG and -IgA responses were not significantly different between QFT-positive and -negative HIV-AC (Table 2). However, 6 of 7 QFT-negative low mitogen responders in HIV-AC were positive for both TBGL-IgG and -IgA assay (data not shown). The TBGL-IgA titers were significantly higher in the TBGL-IgG-positive HIV-AC (*P* = 0.041) (Table 3). In addition, TBGL-IgA-positive HIV-AC had significantly elevated titers of TBGL-IgG (*P* = 0.042), serum IgA (*P* = 0.015), and OPN (*P* = 0.03), (Table 3). Interestingly, the TBGL-IgA-positive proportion was inversely correlated with the CD4+ T-cell counts (*P* = 0.018), and the titers were significantly higher in the HIV-AC with CD4+ T-cell count < 350/*μ*L (HIV-LCD) (*P* = 0.048) (Table 4). Furthermore, in the HIV-AC, a relatively higher proportion of double positive (TBGL-IgG+IgA) responders was found in the HIV-LCD group (29%) than in the HIV-HCD group (CD4+ count ≥ 350/*μ*L) (16%), although the difference was not statistically significant (*P* = 0.32) (Table 4).

Moreover, the IFN-*γ*-nc concentrations were significantly lower in the QFT-negative HIV-AC (*P* = 0.008) (Table 2). No association was observed between the IFN-*γ*-nc concentrations and TBGL-IgG or -IgA titers in any group of HIV-AC (Figure 2). The plasma levels of OPN and leptin were not different between QFT-positive and QTF-negative HIV-AC (Table 2).

### 3.4. Comparison between the Serum Antibodies and TBGL Antibodies

 The TBGL-IgG and -IgA had no correlation with the serum IgG and IgA in HCW and HIV-AC except for the association between the serum IgA levels and the TBGL-IgA titers in HIV-AC (*P* = 0.02) (data not shown). 

### 3.5. Comparison of Biomarkers between HCW and HIV-AC

 The levels of IFN-*γ*-nc (*P* < 0.001) were significantly higher in HCWs than in HIV-AC. However, the titers of TBGL-IgA (*P* = 0.012), but not -IgG, were significantly higher in HIV-AC than in HCWs. Similarly, the serum IgA levels were also higher (*P* = 0.058). The OPN levels were significantly higher (*P* < 0.0001), and the leptin levels were considerably lower (*P* < 0.001) in the HIV-AC compared to the HCWs (Table 1). 

**Figure 3:**
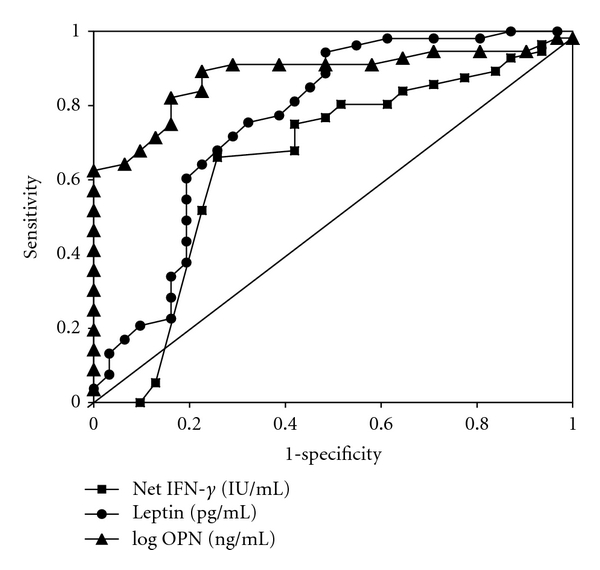
Receiver operating characteristic analysis for comparison of biomarkers between healthcare workers and asymptomatic HIV carriers. The result showed that the OPN plasma levels of OPN (log) exhibited the greatest ability to discriminate HIV from HCW based on the AUC (0.883), followed by leptin (0.763) and net IFN-*γ* (0.648).

ROC curve analysis was used to discriminate HIV from HCW groups using the net IFN-*γ*, leptin, and plasma levels of OPN (log) as biomarkers. As shown in Figure 3, the plasma levels of OPN (log) exhibited the greatest ability to discriminate HIV from HCWs based on the AUC (0.883), followed by leptin (0.763) and net IFN-*γ* (0.648). However, QFT assay as well as TBGL-IgA and -IgG did not show such profiles (data not shown). 

## 4. Discussion

In our data, the application of QFT assay to HCWs in the Philippines demonstrated a high incidence (48%) of LTBI, which was comparable to other already published data in HCWs in TB-endemic developing countries [4]. The increased risk of LTBI among HCWs was confirmed by the recent introduction of IGRA [20, 21]. In our country, a higher incidence of LTBI in HCWs was reported in high-risk groups for TB, such as homeless areas [22], compared to other areas [23]. 

We aimed to clarify the relationship between the TBGL-IgG and -IgA responses and that of IFN-*γ* in the QFT assay in LTBI. The rate of TBGL-IgG positivity was significantly higher in the QFT-positive than QTF-negative group of HCWs. The significant association between the two assay systems indicated by the *κ* value in HCWs demonstrated the TBGL-IgG in LTBI. However, about 30% of QFT-positive populations from among the HCWs lacked TBGL-IgG, and 30% of those of the QFT-negative group have elevated TBGL IgG antibody, and the discordant cases were higher in TBGL-IgA. However, the reasons for such discordances between the two systems in HCWs are not clear. It is possible that the generation of antibody requires larger amounts of antigens than does the generation of T-cell responses. Although associated immunosuppressive conditions were found as risk factors for false-negative QFT responses [24], such cases were excluded from HCWs in our study. 

The mechanism of the synthesis of anti-TDM antibody is not clear, though TDM is known to bind to Mincle (macrophage-inducible C-type lectin) that is present on macrophages [25], and upon the activation, on T cells [26]. It was found that Mincle is specific for the ester linkage of a fatty acid to the trehalose, which explains the strong binding of TDM, but not trehalase-treated TDM, soluble trehalose, or purified mycolate [26]. The conversion of TDM into glucose monomycolate (GMM) upon mycobacterial infection might be the mechanism by which mycobacteria escape from the Mincle-mediated immunity. However, the immune system possesses other tools to monitor and eliminate live mycobacteria through CD1 molecules expressed on the activated macrophages and dendritic cells, which are different from MHC I, II molecules. Recently, GMM but not TDM was demonstrated to interact with CD1b and may induce adaptive immunity [27]. Although it is not known whether the adaptive immune system leads to antibody synthesis, the generated antibody may recognize both TDM and GMM because the two molecules are structurally very similar. 

Interestingly, the IFN-*γ*-nc levels that were observed to have a significant association with the TBGL-IgA titers in LTBI of HCWs. IgA is a typical marker of the mucosal immune response. An elevated serum IgA has been proposed to have a protective role in IFN-*γ*-positive immunocompetent LTBI individuals [28]. Frequent exposure to tubercle bacilli can possibly stimulate the mucosal immune system in TB-endemic countries. It is also known that commensal bacteria on the mucosal surface induce IgA in an NO-dependent manner [29], although it is not known whether MTB in LTBI has a similar effect in lung mucosa. Circulating glycolipid immune complexes might lead to nonspecific stimulation of T cells, but a component of TBGL, TDM, could also enhance the in vivo production of IL-12p40 and IFN-*γ* in mouse model [30]. IgA antibody and IFN-*γ* induce TNF-*α* and NO production, which mediated the inhibitory mechanism for *M. tuberculosis* infection in mouse model [28]. Furthermore, there is strong evidence of a synergic effect between IgA and IFN-*γ* in bactericidal activities against MTB infection [31]. Therefore, the association between anti-TBGL-IgA and IFN-*γ* may indicate protective, mucosal immune activities in LTBI in HCWs. 

In HIV carriers, the QFT-positive responses were significantly lower than in HCWs and were greatly dependent on the high CD4+ T-cell counts in the present study. Much evidence suggests that the baseline CD4+ T-cell count is a determining factor for a positive QFT response in HIV infection [32]. Since HIV infection is a disease of immune deficiency, immune deprivation may be less prominent in relatively young QFT-positive cases because IFN-*γ* could be synthesized properly by stimulation with the appropriate signals. In contrast, the response could be altered in advance immune-deficiency state, as indicated by low CD4+ T-cell counts. Therefore, it is expected that significant numbers of false-negative reactions are present in QFT-negative HIV carriers. The relatively low IFN-*γ* levels by mitogen stimulation in some of the QFT-negative responders also support this possibility. Therefore, for TB diagnosis in advanced immunosuppression, the ratio of the IFN-*γ* response/CD4+ T-cell count Elispot assay was suggested to improve the sensitivity of the assay [33]. 

It is not clear why HIV infection does not diminish the TBGL antibody titers. It is known that the CD-1 presentation pathway persists in patients with HIV, but antiglycolipid antibodies were found to have no relationship with the TST results [34] or bacillary yield [35]. Similarly, we did not find any correlation between the QFT result and anti-TBGL antibodies. It is also possible that concomitant non-TB mycobacterium infection may stimulate the TBGL antibody synthesis in HIV-AC [7]. Significant numbers of HIV carriers have antibodies to TBGL, but we could not confirm if they indicate LTBI or not. 

The increases of serum IgA in advanced HIV infection and of IgG in the early stage were already reported [36]. Although specific antibody titers in HIV infection are decreased by some infectious agents including hepatitis B virus but not in hepatitis A virus, probably because of alterations in the immune systems in advanced HIV infection [37], it is not known whether nonfunctional or functional IgA was synthesized in our cases. The main limitation of the current study is the small number of study subjects and the lack of a follow-up study for estimating the risk of developing active tuberculosis. 

Finally, to determine the correlations between biomarkers in infected states, we evaluated data by ROC curve analysis (Figure 3). In this study, the plasma levels of OPN were most specific to HIV and the levels were not elevated in LTBI HCWs (Figure 3, Table 2). Therefore the levels can be a good marker for active TB in non-HIV individuals, because the OPN is known as a marker of active TB [17]. In HIV-AC, the OPN plasma levels are already elevated as described here, and it was already reported that the levels further increase when they developed active TB [38, 39]. It is also known that interferon-inducible protein-10 (IP-10) and IL-18 were elevated in HIV/TB patients than in HIV patients and suggested to be helpful in monitoring the treatment for patients [38]. All these biomarkers were mainly produced by macrophages, and it was also reported that OPN is synthesized by macrophages as well as CD4+ T cells in HTLV-1-induced lymphoma [40, 41]. 

In this study we noted elevations of anti-TBGL antibody in LTBI in HCWs, but no link between the elevations with LTBI in HIV-AC was confirmed, probably due to the inflammatory conditions in HIV. 

## 5. Conclusion

We have found the elevation of TBGL-IgG titers in LTBI in HCWs. In addition, the association between TBGL-IgA and IFN-*γ* in HCWs was found, and it was hypothesized that the mucosal immunity is involved in LTBI in HCWs. We could not find any relationships between QFT and TBGL in HIV-AC. Low CD4+ cell count was associated with inflammatory conditions as represented by high OPN in HIV-AC, which may be the reason for ambiguous results.

## Figures and Tables

**Table 1:** Demographic and clinical data of study participants.

Variables	HCWs (*n* = 31)	HIV-AC (*n* = 56)	*P*
Demographic data			
Gender: male; *n* (%)	16 (51.6)	55 (96.5)	<0.0001*
Age year; median (range)	35 (19~62)	28 (19~48)	0.03*
Laboratory findings^†^			
Hemoglobin (g/dL)	13.2 ± 2.6	13 ± 1.49	0.36
RBC (million/*μ*L)	4.96 ± 1.6	4.43 ± .55	0.069
WBC (10^3^/*μ*L)	7.5 ± 2.5	5.9 ± 1.9	0.01*
Neutrophil (10^3^/*μ*L)	4.4 ± 2.2	3.3 ± 1.2	0.048*
Lymphocyte (10^3^/*μ*L)	2.4 ± 0.6	2.2 ± 0.9	0.82
Monocyte (/*μ*L)	562 ± 237	338 ± 182	<0.001*
CD4+ T-cell count (/*μ*L)	ND	443 ± 286	NA
QFT assay positive; *n* (%)	15 (48)	13 (23)	0.03*
TBGL-IgG positive; *n* (%)	16 (51)	33 (59)	0.9
TBGL-IgA positive; *n* (%)	6 (19)	16 (29)	0.87
IFN-*γ*-nc (IU/mL)	0.42 ± 0.96	0.13 ± 0.11	<0.001*
TBGL-IgG (U/mL)	3.12 ± 3.36	3.94 ± 6.63	0.14
TBGL-IgA (U/mL)	1.68 ± 2.56	3.1 ± 6.64	0.012*
Serum IgG (mg/dL)	1409 ± 212	1391 ± 224	0.49
Serum IgA (mg/dL)	246 ± 92	319 ± 138	0.058
OPN (ng/mL)	14.4 ± 11	159 ± 191	<0.00001*
Leptin (ng/mL)	18.6 ± 13.9	7.2 ± 5.4	<0.001*

Abbreviations: HCWs, healthcare workers; HIV-AC, newly diagnosed cases of asymptomatic human immune-deficiency virus infection; OPN, osteopontin; ND, not determined; NA, not applicable.

^†^ values were presented as mean ± SD unless indicated otherwise; IFN-*γ*-nc: levels of IFN-*γ*, measured in the nonstimulated QFT-plasma samples; *P* values for statistical differences between HCW and HIV-AC; * significant differences (*P* < 0.05).

**Table 2:** Comparison between QFT-positive and QFT-negative HCWs and HIV-AC.

Variables	HCWs			HIV-AC		
	QFT+ (*n* = 15)	QFT− (*n* = 16)	*P*	QFT+ (*n* = 13)	QFT− (*n* = 43)	*P*
Age; median (range)	45 (21~62)	23.5 (19~48)	0.002*	25 (19~45)	31 (21~35)	0.036*
Gender: male; *n* (%)	7 (46.6)	9 (47.4)	0.43	12 (92.3)	42 (97.67)	0.43
Work duration->10 yrs; *n* (%)	11(73.3)	6 (37.5)	0.098	NA	NA	NA
CD4+ count (/*μ*L); median (range)	ND	ND	NA	611 (148~1466)	356 (13~1125)	0.012*
TBGL-IgG positive; *n* (%)	11 (73)	5 (31)	0.02*	8 (61.5)	25 (58.13)	0.545
TBGL-IgA positive; *n* (%)	5 (33)	1 (6)	0.072	3 (23)	13 (30)	0.415
TBGL-IgG+IgA positive; *n* (%)	4 (27)	0 (0)	0.043*	2(15.4)	10 (23.3)	0.42
IFN-*γ*-nc (IU/mL)^†^	0.3 ± 0.4	0.2 ± 0.13	0.9	0.21 ± 0.17	0.1 ± 0.07	0.0087*
Serum IgG (mg/dL)^†^	1450 ± 188	1368 ± 235	0.2	1306 ± 207	1414 ± 249	0.5
Serum IgA (mg/dL)^†^	268 ± 81	225 ± 101	0.32	330 ± 130	312 ± 138	0.68
OPN (ng/mL)^†^	14.5 ± 11.2	14.2 ± 11.2	0.87	115.4 ± 130	173.2 ± 203	0.43
Leptin (ng/mL)^†^	21.3 ± 13.3	15.9 ± 14.3	0.25	6.46 ± 4.12	7.448 ± 5.68	0.24

Abbreviations: HCWs, healthcare workers; HIV-AC, newly diagnosed cases of asymptomatic human immune-deficiency virus infection; OPN, osteopontin; ND, not determined; NA, not applicable.

^†^mean ± SD; IFN-*γ*-nc: levels of IFN-*γ*, measured in the nonstimulated QFT-plasma samples; *P* values for statistical differences between QFT-positive and QTF-negative groups; *significant differences (*P* < 0.05).

**Table 3:** Comparison between TBGL-IgG or TBGL-IgA-positive and -negative HIV-AC.

Variables	TBGL-IgG			TBGL-IgA		
	Positive (*n* = 33)	Negative (*n* = 23)	*P*	Positive (*n* = 16)	Negative (*n* = 40)	*P*
Age; median (range)	28 (19~48)	30 (19~41)	0.18	31.5 (19~48)	28 (19~45)	0.038*
Gender: male; *n* (%)	33 (100)	21 (91.3)	0.43	16 (100)	38 (95)	1
CD4 count (/*μ*L); mean (range)	436 (13~1466)	450 (60~851)	0.45	346 (46~1125)	480 (13~1466)	0.06
QFT positive; *n* (%)	8 (24.2)	5 (21.7)	0.545	3 (19)	10 (25)	0.45
TBGL-IgA positive; *n* (%)	12 (36.4)	4 (17.4)	0.1	—	—	—
TBGL-IgG positive; *n* (%)	—	—	—	12 (75)	21 (52.5)	0.14
IFN-*γ*-nc (IU/mL)^ †^	0.13 ± 0.09	0.1 ± 0.05	0.4	0.12 ± 0.09	0.12 ± 0.07	0.9
TBGL-IgA (U/mL)^†^	4.36 ± 8.4	1.28 ± 1.21	0.041*	—	—	
TBGL-IgG (U/mL)^†^	—	—		7.5 ± 11.6	2.5 ± 1.5	0.042*
Serum IgG (mg/dL)^†^	1439 ± 277	1515 ± 677	0.5	1615 ± 404	1355 ± 135	0.46
Serum IgA (mg/dL)^†^	277 ± 95	279 ± 74	0.37	410 ± 165	313 ± 138	0.015*
OPN (ng/mL)^†^	176.3 ± 199.9	136 ± 172.5	0.67	280 ± 275	115 ± 109.7	0.03*
Leptin (ng/mL)^†^	7.33 ± 6.16	7.18 ± 4.12	0.68	7.33 ± 6.16	7.18 ± 4.12	0.07

Abbreviations: HIV-AC, newly diagnosed cases of asymptomatic human immune-deficiency virus infection; OPN, osteopontin.

^†^mean ± SD; IFN-*γ*-nc: levels of IFN-*γ*, measured in the nonstimulated QFT-plasma samples; *P* for statistical differences between QFT-positive and QTF-negative groups; *significant differences (*P* < 0.05).

**Table 4:** Comparison between HIV-AC with high^§^ and low^‡^ CD4+ T-cell count.

Variables	CD4+ high^§^ (*n* = 32)	CD4+ low^‡^ (*n* = 24)	*P* value^¶^
Age; mean (range)	25.5 (19~45)	25 (22~48)	0.018*
Gender: male; *n* (%)	31 (97)	23 (98)	1.0
CD4+ count (/*μ*L); median (range)	618 (356~1466)	201 (13~349)	<0.001*
QFT-positive; *n* (%)	10 (31)	3 (12.5)	0.12
TBGL-IgG positive; *n* (%)	16 (50)	16 (67)	0.27
TBGL-IgA positive; *n* (%)	5 (16)	11 (46)	0.018*
TBGL-IgG+ IgA positive; *n* (%)	5 (16)	7 (29)	0.32
IFN-*γ*-nc (IU/mL)	0.14 ± 0.12	0.13 ± 0.09	0.9
TBGL-IgG (U/mL)^†^	4.6 ± 8.4	3 ± 2.8	0.59
TBGL-IgA (U/mL)^†^	1.55 ± 2	5.16 ± 9.6	0.048*
Serum IgG (mg/dL)^†^	1352 ± 185	1549 ± 380	0.5
Serum IgA (mg/dL)^†^	265 ± 89	423 ± 149	<0.001*
OPN (ng/mL)^†^	119 ± 126	214 ± 246	0.19
Leptin (ng/mL)^†^	7.7 ± 6	6.6 ± 4.9	0.5

Abbreviation: HIV-AC, newly diagnosed cases of asymptomatic human immune-deficiency virus infection; OPN: osteopontin.

^§^High: CD4+ T cell count ≥350/*μ*L; ^‡^low: CD4+ T-cell count <350/*μ*L; ^†^mean±SD; IFN-*γ*-nc: levels of IFN-*γ*, measured in the non-stimulated QFT-plasma samples; *P* values for statistical differences between QFT-positive and QTF-negative groups; *significant differences (*P* < 0.05).
